# Coarse-Grained Simulations of Aqueous Thermoresponsive Polyethers

**DOI:** 10.3390/polym10050475

**Published:** 2018-04-27

**Authors:** Bryan Raubenolt, Gaurav Gyawali, Wenwen Tang, Katy S. Wong, Steven W. Rick

**Affiliations:** 1 Department of Chemistry, University of New Orleans, New Orleans, LA 70148, USA; braubeno@my.uno.edu (B.R.); ggyawali@my.uno.edu (G.G.); 2 Benjamin Franklin High School, New Orleans, LA 70122, USA; wtang43@gmail.com; 3 Department of Chemical and Biomolecular Engineering, Tulane University, New Orleans, LA 70118, USA; kwong5@tulane.edu

**Keywords:** coarse-grain, simulations, polymers, polyethylene oxide, thermoresponsive

## Abstract

Thermoresponsive polymers can change structure or solubility as a function of temperature. Block co-polymers of polyethers have a response that depends on polymer molecular weight and co-polymer composition. A coarse-grained model for aqueous polyethers is developed and applied to polyethylene oxide and polyethylene oxide-polypropylene oxide-polyethylene oxide triblock co-polymers. In this model, no interaction sites on hydrogen atoms are included, no Coulombic interactions are present, and all interactions are short-ranged, treated with a combination of two- and three-body terms. Our simulations find that The triblock co-polymers tend to associate at temperatures above 350 K. The aggregation is stabilized by contact between The hydrophobic methyl groups on The propylene oxide monomers and involves a large, favorable change in entropy.

## 1. Introduction

**Figure 1:**
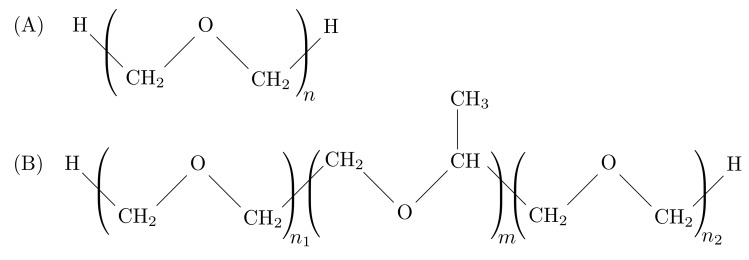
Structures of PEO (**A**) and the PEO-PPO-PEO (**B**) triblock copolymers.

Polyethers, including polyethylene oxide (PEO), polyethylene glycol (PEG), and polypropylene oxide (PPO), are versatile polymers with a wide range of applications in chemistry, biochemistry, materials, and manufacturing [1]. The amphiphilic nature of the polymers are evident in the complex phase behavior. PEO is water soluable at room temperature, at all concentrations and all polymer lengths, but at much higher temperatures, there is a miscibility gap and the polymer solution phase separates into two phases. This lower critical solution temperature (LCST), or cloud point, occurs at a temperature dependent on concentration and length [2]. The more hydrophobic triblock PEO-PPO-PEO polymers, with composition (EO)${}_{n1}$-(PO)${}_{m}$-(EO)${}_{n2}$ (Figure 1), have lower cloud points that can be near room temperature [2,3,4]. There are a variety of such polymers available under the commercial names Pluronic, Poloxamers, and Tetronics. The solvation of PEO and the Pluronic polymers has a large unfavorable entropic contribution, so the free energy becomes less favorable as temperature increases. By tuning the strengths of the unfavorable entropy and favorable enthalpy, a polymer with a specific LCST, or cloud point, can be designed. This tuneability makes Pluronic polymers attractive polymers for therapeutic applications, including drug delivery and pharmaceutical formulation [4]. Understanding the thermal response of the polymers, in terms of the structural changes involved and roles of entropy and enthalpy, would aid in the rational design of these materials.

Accurate molecular models for polyethers need to get the correct amount of hydrophilicity and also ideally be computationally efficient for large scale simulations. Given the importance of PEO, and related ether compounds, a number of atomistic potentials have been developed [5,6,7,8]. There are also coarse-grained models, in which a single or multiple polymer unit is reduced to a single interaction site [9,10,11,12,13,14,15,16]. It is a challenge for molecular simulations to study the thermal response of polymer aggregation, both because long sampling times might be required and it is a strong test of the force field to get the balance of water-water, water-polymer, and polymer-polymer interactions correct. Due to this challenge, few simulation studies have directly found an LCST [17]. One study using a CG model examined the formation of micelles of Pluronic polymers, but did not examine the temperature response [10]. Another study, with temperature dependent CG models, looked at the thermal response of a single PEO chain [16].

This manuscript describes a new model for aqueous polyethers, based on the Stillinger-Weber (SW) model [18], which gains efficiency through the elimination of hydrogen interaction sites, including those on polar hydrogens, and through a reduction in the range of the potential, which includes the elimination of all long-ranged Coulombic interactions. This method has proved effective to simulating water [19,20,21], aqueous salt solutions [22], and aqueous [23,24] and liquid [24] alkanes. There are other approaches which result in efficient one-site water models [25,26], using a point dipole [26] or higher order multipoles [25]. The Stillinger-Weber model was chosen because it builds on earlier work and since its interactions are shorter-ranged, it has the potential to be even more efficient than models which include some treatment of electrostatics. In addition, by only removing hydrogen atoms, and not coarse-graining on a larger length-scale, there is not a significant loss in degree-of-freedom of the polymer. Except for the terminal methyl groups, The hydrogen atoms are highly constrained, which is why united atoms models can be very accurate over ranges of temperatures [27,28,29] and hydrogen atoms can be constrained, using SHAKE and RATTLE, in all-atom models without significant change in the resulting properties. For water, the hydrogens do represent important degrees-of-freedom, but the monatomic water (mW) model, using the SW potential, reproduces the properties of water over a range of temperatures [19]. In addition, the mW model can capture the hydrophobic effects, including the methane pair potential of mean-force [23]. The surface tension of a water/alkane interface [24,30], and the melting temperature of methane hydrate clathrates [30]. These results suggest that SW potentials can accurately and efficiently model aqueous polyethers. Model parameters are optimized to reproduce properties of 1,2-dimethoxyethane (DME) as a pure liquid and in mixtures with water, as well as aqueous PEO. Using the validated potentials, simulations of the Pluronic polymer L42, are carried out. L42 has an LCST in a 1 weight percent aqueous solution equal to 37 ${}^{\circ }$C [3]. This will be compared to simulations of EO${}_{30}$, which does not have an LCST below 100 ${}^{\circ }$C. Simulations at different temperatures will study the aggregation behavior of L42 and EO${}_{30}$.

## 2. Materials and Methods

The potential energy of the system is modeled using the Stillinger-Weber potential [18] which is a combination of two- and three-body interactions,

(1) $$E=\sum_{i}\sum_{j>i}\phi_{2}\left(r_{ij}\right)+\sum_{i}\sum_{j\neq i}\sum_{k>j}\phi_{3}\left(r_{ij},r_{ik},\theta_{jik}\right)$$

The two-body term is given by

(2) $$\phi_{2}\left(r_{ij}\right)=\left\{\begin{array}{cc} A\epsilon_{ij}\left[B{\left(\sigma_{ij}/r_{ij}\right)}^{p}-{\left(\sigma_{ij}/r_{ij}\right)}^{q}\right]\exp\left(\sigma_{ij}/\left(r_{ij}-a_{ij}\sigma_{ij}\right)\right) & \mathrm{if}r_{ij}<a_{ij}\sigma_{ij}\\ 0 & \mathrm{if}r_{ij}>a_{ij}\sigma_{ij} \end{array}\right.$$

with *p* equals 4 and *q* equals 0. The two body term is characterized by a well-depth, ϵ, and a length scale, σ. The three body term is given by

(3) $$\phi_{3}\left(r_{ij},r_{ik},\theta_{jik}\right)=\left\{\begin{array}{cc} \lambda_{ijk}\epsilon_{ijk}{\left[\cos\theta_{jik}-\cos\theta_{0}\right]}^{2}\exp\left(\gamma \sigma_{ij}/\left(r_{ij}-a_{ij}\sigma_{ij}\right)\right)\exp\left(\gamma \sigma_{ik}/\left(r_{ik}-a_{ik}\sigma_{ik}\right)\right) & \mathrm{if}r_{ij}<a_{ij}\sigma_{ij}\mathrm{and}r_{ik}<a_{ik}\sigma_{ik}\\ 0 & \mathrm{otherwise} \end{array}\right.$$

and acts between central particle *i* and its two neighbors *j* and *k*, where $\theta_{jik}$ is The angle between those three particles, $\theta_{0}$ is the target value of that angle, and λ scales the strength of $\phi_{3}$. Rigorously, both $\phi_{2}$ and $\phi_{3}$ go to zero beyond a length $a_{ij}\sigma_{ij}$, reducing the range of the potential, unlike commonly used potentials, like Lennard-Jones or Coulombic interactions. As in previous studies [19,22,24,30], some parameters are fixed (*A* = 7.049556277, *B* = 0.6022245583, and γ = 1.20) and others (ϵ, σ, λ, $\theta_{0}$, and, in one case, a) treated as adjustable. Torsional energies used the Fourier form

(4) $$E\left(\phi \right)=\sum_{n=0}^{N}k_{n}\left[1+\cos\left(n\phi -\phi_{n}\right)\right]$$

The angle $\phi_{n}$ is 0, except for *n* = 2, for which it is 180${}^{\circ }$. The force constants are, for the C–O–C–C bond, *k*${}_{1}$ = 0.90 kcal/mol, *k*${}_{2}$ = −0.3240 kcal/mol, and *k*${}_{3}$ = 1.1093 kcal/mol and, for O–C–C–O, *k*${}_{0}$ = 0.50 kcal/mol, *k*${}_{0}$ = −0.400 kcal/mol, and *k*${}_{2}$ = 2.000 kcal/mol. Other terms not given are zero. For the bond stretch, $E=k_{b}{\left(r-r_{0}\right)}^{2}$, and bond angle terms, $E=k_{\theta }{\left(\theta -\theta_{0}\right)}^{2}$, we use the TraPPE-UA values [31] ($k_{b}$ = 260.40 kcal/mol Å${}^{-2}$, $r_{0}$ = 1.54 Å for C–C bonds, $k_{b}$ = 320.45 kcal/mol Å${}^{-2}$, $r_{0}$ = 1.41 Å for C–O bonds, $k_{\theta }$ = 50.0 kcal/mol, $\theta_{0}$ = 112 for C–C–O angles, and $k_{\theta }$ = 62.2 kcal/mol, $\theta_{0}$ = 112 for C–C–O angles). No 1–4 interactions are included in the potential. The optimized parameters are given on Table 1 and Table 2. As in a previous study [24], The lack of combining rules allows for the optimization process to be divided in two parts, first for the solute-solute interactions and then for the solute-water interactions. This process involved systematically varying the parameters to optimize the properties listed in the Results section. The present system has only three different atom types so the optimization is fairly straightforward. The parameterization of more complex systems would benefit from automated parameter optimization [32,33].

The enthalpy of vaporization is found from $\Delta H_{vap}=\langle E_{g}\rangle -\langle E_{l}\rangle +RT$, where $\langle E_{g}\rangle$ is the gas-phase energy and $\langle E_{l}\rangle$ is the liquid-phase energy. The surface tension is found from $\gamma =\frac{L_{z}}{2}\left[P_{zz}-\left(P_{xx}+P_{yy}\right)/2)\right]$, where and *P*${}_{\alpha \alpha }$ is the diagonal pressure tensor, *z* is the direction perpendicular to The interface, and *L*${}_{z}$ is the box length in that direction. The free energy of aqueous solvation, $\Delta G_{solv}$, is found using finite difference thermodynamic integration (FDTI) [34], and a soft core potential to avoid singularities [35]. The subroutines necessary to implement TI in LAMMPS with The SW potentials were added by our group and these routines, along with sample input files, are available on github [36]. The enthalpy of solvation was found from $\Delta H_{solv}={\langle E\left(DME\right)\rangle }_{aq}-{\langle E\left(DME\right)\rangle }_{gp}-{\langle E\left(water\right)\rangle }_{aq}+P\Delta V$, where ${\langle E\left(DME\right)\rangle }_{aq}$ is the energy of aqueous DME (with 1 DME molecule and 256 water molecules), ${\langle E\left(DME\right)\rangle }_{gp}$ is the energy of 1 DME molecule in the gas-phase, ${\langle E\left(water\right)\rangle }_{aq}$ is the energy of 256 water molecules, *P* is the pressure, and Δ*V* is the volume change between the solution and pure water. A relatively small system size was chosen to minimize the noise in the energies. The entropy of solvation was found from $\Delta S_{solv}=\left(\Delta H_{solv}-\Delta G_{solv}\right)/T$.

We take (EO)${}_{4}$-(PO)${}_{22}$-(EO)${}_{4}$ as the chemical structure L42, which is consistent with its reported molecular weight (1630 g/mol) and EO weight percent (20) [3]. For simulations of the Pluronic polymer, parameters for the propylene oxide methyl group were taken from the alkane force field previously developed [24]. Those parameters, denoted CH${}_{3}$(A), are given in Table 1. For the interactions between this methyl atom and the other polymer atoms, The Lorentz-Berthelot combining rules were used, so that $\epsilon_{ij}={\left(\epsilon_{ii}\epsilon_{jj}\right)}^{1/2}$ and $\sigma_{ij}=\left(\sigma_{ii}+\sigma_{jj}\right)/2$, and The parameter a was set equal to 1.8 Å. These combining rules were used only for the interactions between this CH${}_{3}$ group and the other polymer atoms. All other two-body parameters are as specified in Table 1. No three-body interactions were used for this methyl group. The polymer was constructed to be atactic, with a stereochemistry chosen randomly for the PO units.

All CG simulations used the LAMMPS [37] program with a 5 fs time step in the isothermal-isobaric (TPN) ensemble. Temperature and pressure were controlled with a Nosé-Hoover thermostat, with a 100 fs damping constant for temperature and a 1000 ps damping constant for pressure. The CG liquid DME simulations used 486 molecules. The water/DME mixtures used a similar number of molecules, adjusted to give the specified mole fraction. Simulations with one polymer of various lengths used 2479 water molecules. The liquid and aqueous DME systems were run for about 2 ns and the polymers for were run to 100 ns. To examine aggregation, simulations of the (EO)${}_{30}$ and the L42 polymers in solution were carried out. These simulations used 2 polymers with 16,500 water molecules, corresponding to 1 weight percent solution, and were simulated for 1 microsecond. At this concentration, L42 has a cloud point temperature of 37 ${}^{\circ }$C [3]. The all-atom and united-atom simulations used for parameter optimization were done with GROMACS [38]. To test the efficiency of the CG model, we ran additional all-atom simulation using LAMMPS. These simulations were for (EO)${}_{30}$, using the OPLS-AA model [39], and 2479 water molecules, using the SPC/E model [40]. These simulations used particle mesh Ewald and a 1 fs time step. For the same number of heavy atoms and the same simulation time, the CG simulations run about a factor of 10 times faster than the all-atom simulations. This is at least an order of magnitude [23] less than the predicted gains in speed due to the larger time step, reduced range of interactions, and fewer interaction sites. The implementation of the SW potential does not appear to be as optimized as the much more standard, all-atom models and it may be that further optimization would be beneficial. Still, the speed-ups we do get allow us to carry our simulations of relatively large systems (the L42 dimer in water has 16,724 heavy atoms) for a microsecond.

## 3. Results

### 3.1. Properties of The Optimized Model for Linear Ethers

The CG model uses the Stillinger-Weber (SW) potential [18], as described in the Methods section. The parameters for the model between ether molecules were chosen to optimize the experimental density [41], enthalpy of vaporization [42], and surface tension [43] for liquid 1,2-dimethoxyethane (DME) at 1 atm and 298 K. The oxygen-CH${}_{2}$ radial distribution function from the modified TraPPE-UA model [44] was used to parameterize against as well. For the water-ether interactions, the solvation free energy and enthalpy [45], and the DME/water density as a function of mole fraction [41], as well as calculated oxygen-water radial distribution functions from both the modified TraPPE-UA model [44] and Smith, et al. model were used.

The liquid state properties are well-reproduced by the model (Table 3). Previous studies using the SW potential have also found accurate surface tensions for pure water [19], pure liquid alkanes [24], and the alkane/water interface [24]. The model, despite having no hydrogen interaction sites and no long-ranged interactions, is able to reproduce the surface tensions as well as all-atom models.

**Figure 2:**
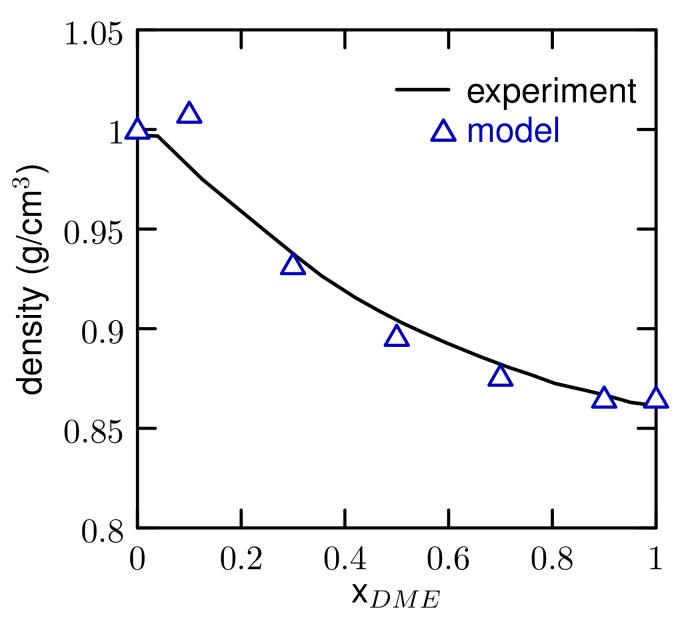
Density of The water/DME mixtures.

The solvation free energy, enthalpy, and entropy of DME in water are in good agreement with experiment. The large unfavorable entropy of solvation is captured by the model, which is important for the thermal response of the polyethers. The density as a function of mole fraction for DME/water mixtures (Figure 2) agrees with experiment [41]. The value at a small mole fraction (X${}_{DME}$ = 0.1) is slightly overestimated, perhaps because the hydration layer around DME is overly dense. As the DME concentration increases, the deviation from experiment decreases. The density versus composition is very sensitive the balance of the interactions between all three pairs (water-water, DME-DME, DME-water) and the agreement with experiments provides some confidence that the models have this balance about right. The error estimates in the densities are all about 0.002 g/cm${}^{3}$, smaller than the size of the symbols in Figure 2.

**Figure 3:**
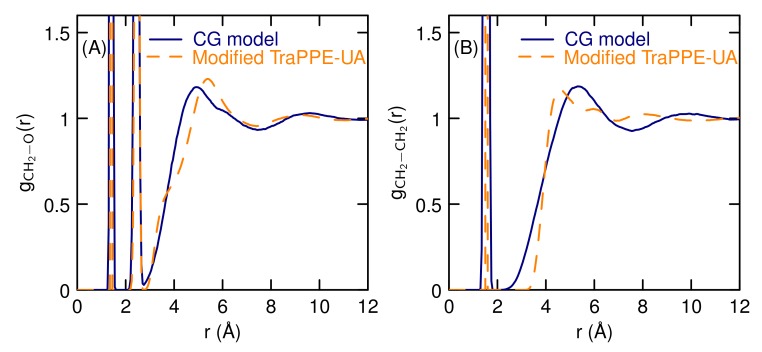
Liquid DME (**A**) oxygen-carbon (**B**) carbon-carbon radial distribution functions.

**Figure 4:**
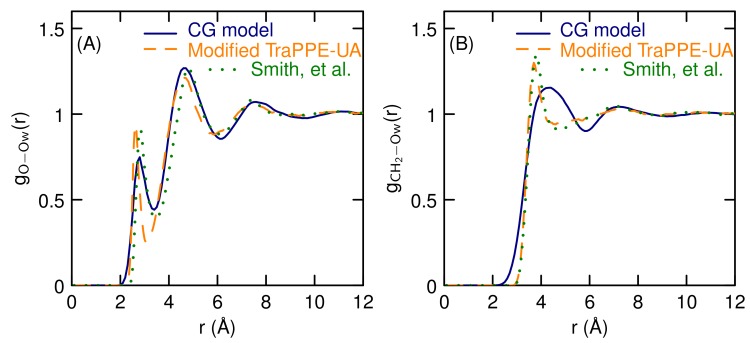
(**A**) Water oxygen-DME oxygen and (**B**) water oxgyen-DME carbon radial distribution functions.

The radial distribution functions for liquid DME show similar structure to the modified TraPPE-UA model of Fischer, et al. [44], as can be seen in Figure 3. The radial distribution functions do not have much structure but the CG model has a first peak position and height about the same as the conventional model. Figure 4 shows the DME oxygen/water oxygen and DME CH${}_{2}$ carbon/water radial distribution functions, comparing the present model to both the modified TraPPE-UA/TIP4P-Ew [44,46] and Smith, et al./TIP4P [47] models. There are some differences between the published models for the DME oxygen/water oxygen radial distribution functions, both in terms of the position and the height of the first peak. Our model gives two water molecules in this peak, in agreement with the Smith, et al. results. The modified TraPPE-UA model gives a value closer to one. We did not use the temperature dependence of any property in the parameterization process, but for the purposes of this study, capturing the effects of temperature is important. These radial distribution functions have a very weak temperature dependence, from either the CG or other models (data not shown). For pure DME, g${}_{CH_{2}-O}$(r) and g${}_{CH_{2}-CH_{2}}$(r) are virtually the same at 300 and 350 K. For the aqueous pair correlation functions, the peaks stay in same position but decrease by a small amount. For example, the first peak for g${}_{O-O_{w}}$(r) decreases from 0.82 to 0.70 for the CG model as the temperature increases from 300 to 350 K. For the Smith, et al./TIP4P [47] model, the first peak decreases from 0.91 to 0.86. These changes have negligible effects on the coordination numbers. So the model does reproduce the weak temperature dependance of the other models over the temperature range of interest.

**Figure 5:**
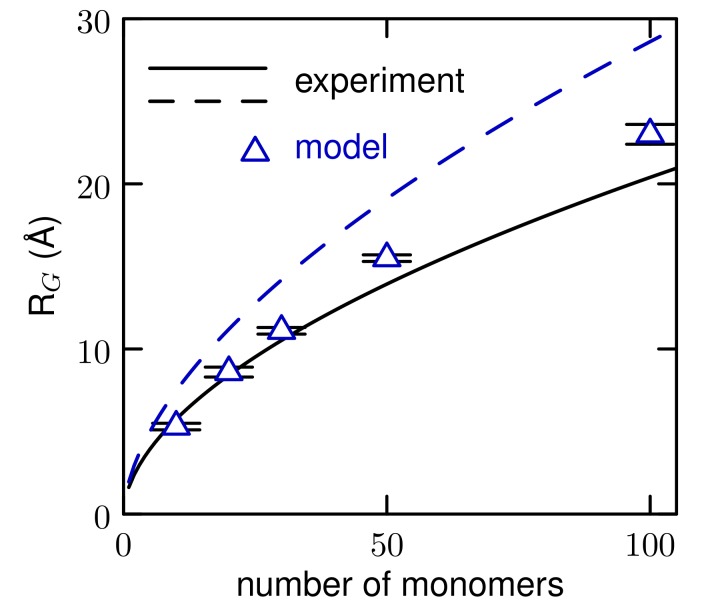
Radius of gyration of PEO as a function of length.

The radius of gyration, *R*${}_{g}$, of the PEO polymers of various lengths is shown in Figure 5. The light scattering experiments of Kawaguchi, et al. [48], (solid line) and Devanand and Selser [49] (dashed line) find slightly different results. Our results are close to both, as are both atomistic [44] and other CG models [14,15]. Experimentally, *R*${}_{g}$ scales as N${}^{\alpha }$, where N is The number of monomers. Kawaguchi, et al. report an exponent of 0.550 and Devanand and Selser find 0.583. Our results give an exponent of 0.56.

Overall, the model does well in reproducing thermodynamical properties including density and surface tension of pure DME. Aqueous properties, including the free energy and entropy of solvation, the density of DME/water mixtures, and the radius of gyration of The PEO in water, are also well-reproduced.

### 3.2. Simulations of (EO)${}_{30}$ and L42

**Figure 6:**
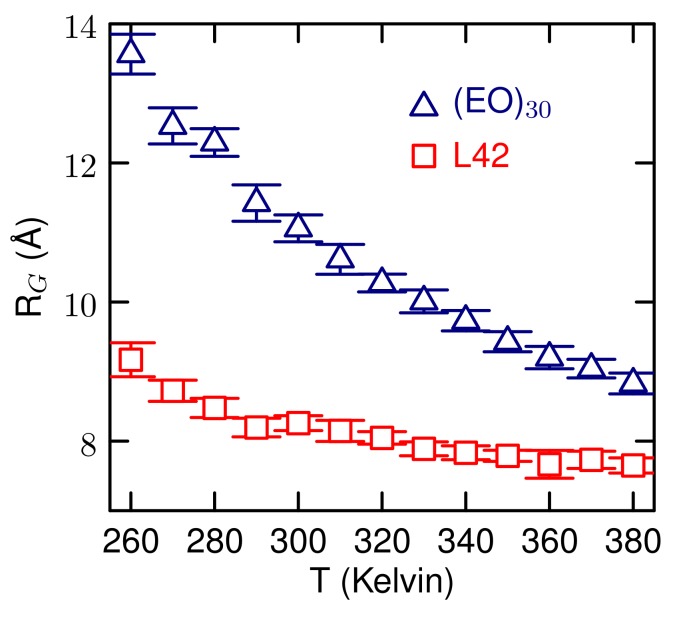
Radius of gyration for a single polymer as a function of temperature.

Simulations of a single polymer in water over a range of temperatures were carried out. The radius of gyration is shown in Figure 6. From The *R*${}_{G}$, there is no sign of a transition from an extended to a compact form, just a gradual decrease in size as temperature increases. The L42 polymer is more compact in solution than (EO)${}_{30}$. Simulations studies of PEO using CG models find a similar small decrease in *R*${}_{G}$ for shorter chains and only chains above about 500 monomer units show signs of an extended to compact structure [16].

**Figure 7:**
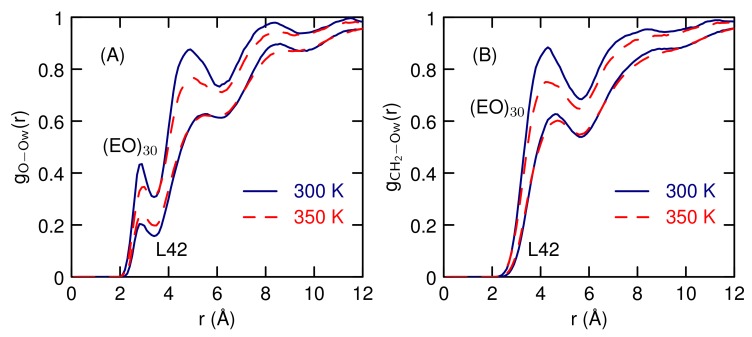
Water oxygen-polymer oxygen (**A**) and water oxygen-polymer CH${}_{2}$ (**B**) radial distribution functions at 300 and 350 K for a single (EO)${}_{30}$ (The top two curves) and L42 (The bottom two curves) polymer.

The radial distribution functions between the polymers and water (Figure 7) reveals that L42 has less water contact than (EO)${}_{30}$ and both have smaller peaks than DME in water. As temperature increases, the peaks decrease, suggesting that the decrease in size indicated by the smaller *R*${}_{G}$ is due to a decrease in the amount of water near the polymer. The biggest decrease, for both (EO)${}_{30}$ and L42, is near the CH${}_{2}$ group (Figure 7B). There is 1.3 water molecules in the nearest neighbor peak for g${}_{O-O_{W}}$(r) for (EO)${}_{30}$ at 300 K, which decreases to 1.1 at 350 K. This number is consistent with the results of all atom simulations [50,51]. For L42, the first peak of g${}_{O-O_{W}}$(r) for L42 which increases slightly and first peak gives 0.6 waters at 300 K and increases to 0.7 at 350 K. For L42 there also a similar decrease in the nearest neighbor peak between the PPO methyl group and water (from 0.71 to 0.66, data not shown). Overall, the changes in the water-polymer radial distribution functions are small, as are the changes in *R*${}_{G}$.

**Figure 8:**
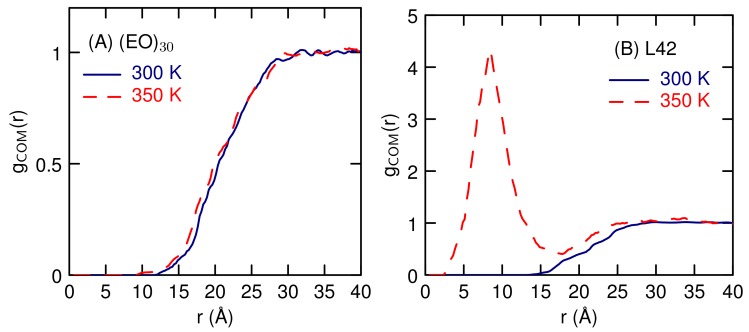
Radial distribution functions between the polymer centers-of-mass at different temperatures for aqueous dimers of (**A**) EO${}_{30}$ and (**B**) L42.

Simulations of two polymer molecules, at a concentration of 1 weight percent, find differing amounts of aggregation for each polymers as a function of temperature. The radial distribution function between the centers-of-mass, $g_{COM}$, for the (EO)${}_{30}$ polymers (Figure 8A) shows very little structure. There is no tendency to form an aggregate pair and there is very little difference between the two temperatures 300 and 350 K. There is not significant probability to find the polymers at a distance less than 20 Å, about twice the radius of gyration. For L42, at 300 K, $g_{COM}$ is similar to the (EO)${}_{30}$ result, but at 350 K there is a clear tendency to associate, with a large peak at 8 Å. This corresponds to a distance much less that twice *R*${}_{G}$. During the 1 microsecond simulation, the contact pair forms and dissociates a number of times, forming about once every 0.1 microseconds.

**Figure 9:**
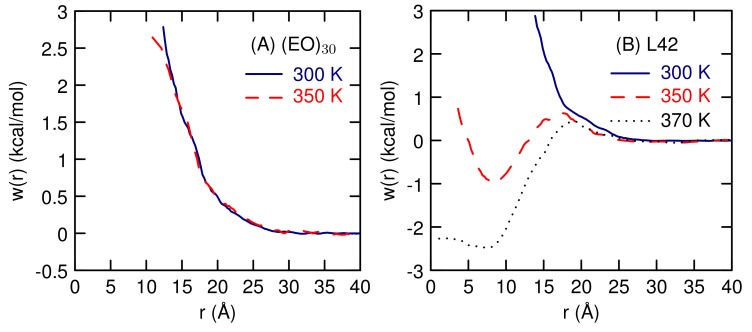
The potential of mean-force between The polymer centers-of-mass at different temperatures for aqueous dimers of (**A**) EO${}_{30}$ and (**B**) L42.

The potential of mean-force, *w*(*r*), between the two polymers can be found from the radial distribution functions using $w\left(r\right)=-k_{B}Tlng_{COM}\left(r\right)$, where $k_{B}$ is Boltzmann’s constant. For (EO)${}_{30}$ and L42 at 300 K, w(r) looks like a single exponential decay, with no minima corresponding to a contact pair (Figure 9). (Fitting to w(r) = *A*e${}^{-r/\alpha }$, with an exponential decay constant, α, of 32 Å for (EO)${}_{30}$ and 36 Å for L42 at 300 K.) At 350 K, there is a contact pair minimum. Simulations of the L42 dimer were carried at an additional temperature of 370 K, so that the temperature dependence of the contact pair minimum can be determined. The contact pair minimum gets deeper at the higher temperature. At both 350 and 370 K, the two polymers are found at close distances, less than twice *R*${}_{G}$, which means in the contact pair the polymers are not spherical.

**Figure 10:**
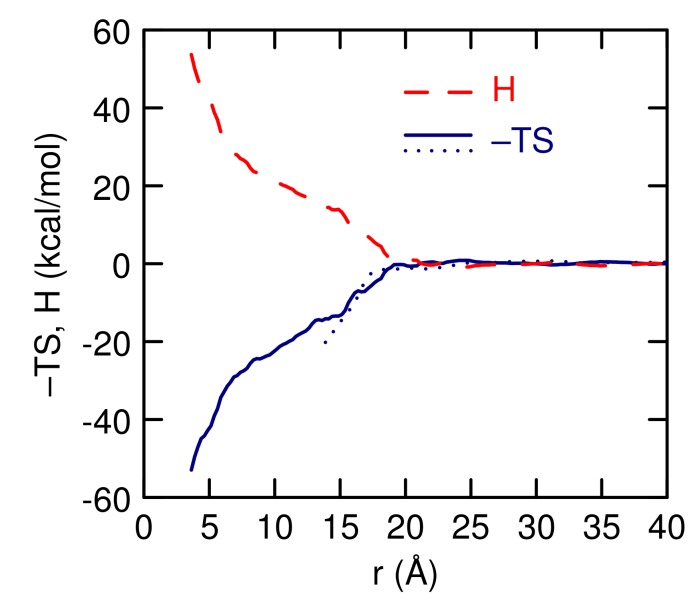
The enthalpy, H, (red dashed lined), and entropy, −S, (blue solid and dotted lines) as a function of The distance between The polymer centers-of-mass at 350 K. The solid line determines S from w(r) at 350 and 370 K and the dotted line uses 300 and 350 K.

From The temperature dependence of w(r), The entropy can be found, from

(5) $$S\left(r\right)=-\left(w_{T_{2}}\left(r\right)-w_{T_{1}}\left(r\right)\right)/\left(T_{2}-T_{1}\right)$$

where $w_{T_{j}}\left(r\right)$ is the PMF at temperature *T*${}_{j}$. The enthalpy can be found from H(r)=w(r)+TS(r). This expression for the entropy assumes w(r) is linear with respect to temperature , or, equivalently, S(r) independent of temperature, over the temperature range. The entropy, calculated using w(r) at 300 and 350 K and using w(r) at 350 and 370 K, show good agreement with each other over The range of distances for which w(r) at 300 K is determined, starting at 14 Å (Figure 10). This suggests that the assumption of temperature independence of S(r) is reasonable. There is a large entropic stabilization of the contact pair. The minimum of w(r), at 350 K, occurs at 8.4 Å, and at that distance −TS(r) is −25 kcal/mol. The enthalpy is large and positive.

**Figure 11:**
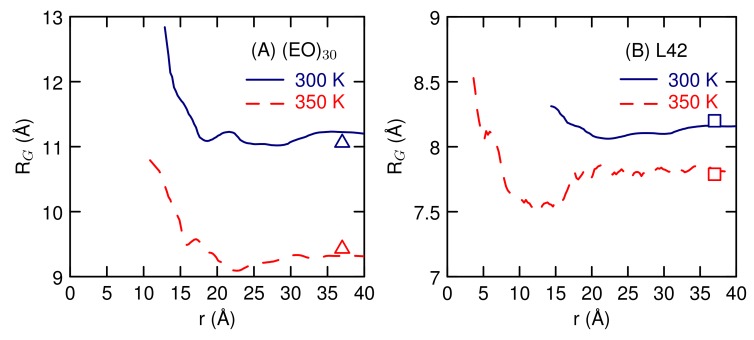
The single-polymer radius of gyration as a function of the distance between the centers-of-mass of the two polymers for (**A**) EO${}_{30}$ and (**B**) L42. The symbols give the corresponding *R*${}_{G}$ for the isolated polymer.

The radius of gyration of the polymer as a function of the separation between the centers-of-mass (Figure 11) shows that the (EO)${}_{30}$ polymers get larger when they are in contact. When they are separated, the radius of gyration has the same value it has in the single polymer simulation (the symbols in Figure 11). Small angle neutron scattering (SANS) experiments of PEG find that *R*${}_{G}$ decreases with increasing concentration [52,53]. Our results suggest that close contact between PEO polymers increases *R*${}_{G}$. Other simulation results do not see a reduction in *R*${}_{G}$ as well [11,14,15]. The L42 polymers at 300 K also get larger, by a small amount, when they are in contact. At 350 K, the L42 polymers have a smaller *R*${}_{G}$ when in contact.

**Figure 12:**
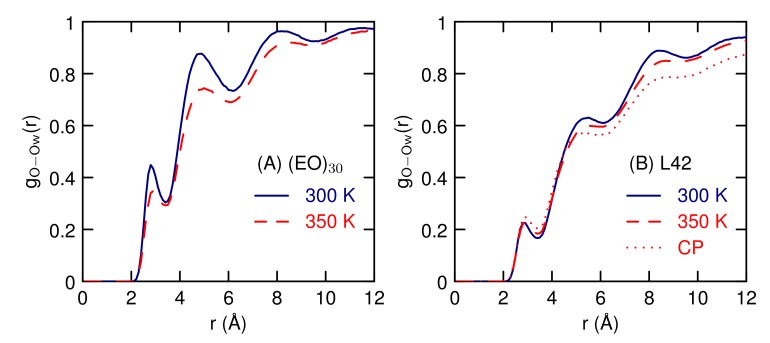
Water oxygen-polymer oxygen radial distribution function at different temperatures for dimers of (**A**) EO${}_{30}$ and (**B**) L42. For L42, The results for The contact pair (CP) at 350 K are also shown.

The water structure around the polymers, as given by the radial distribution function between the polymer and water oxygens (Figure 12) is similar to the results of the single polymer (Figure 7) showing a weak temperature dependence. For (EO)${}_{30}$, the hydrogen-bonding peak, at 2.8 Å, indicates that there is one nearest-neighbor water molecule near the oxygen atom, at 300 K. The peak heights decrease slightly with temperature. The water structure around L42 is significantly reduced relative to (EO)${}_{30}$, with a water coordination number around 0.5. Structures of the contact pair, at 350 K, were used to analyze the solvent structure (the CP curve on Figure 12B). The contact pair is taken to be any pair that has a center-of-mass distance less than 17 Å. This shows that the first peak is about the same, whether or not the polymers are in contact, so contact does not displace nearest neighbor water molecules.

**Figure 13:**
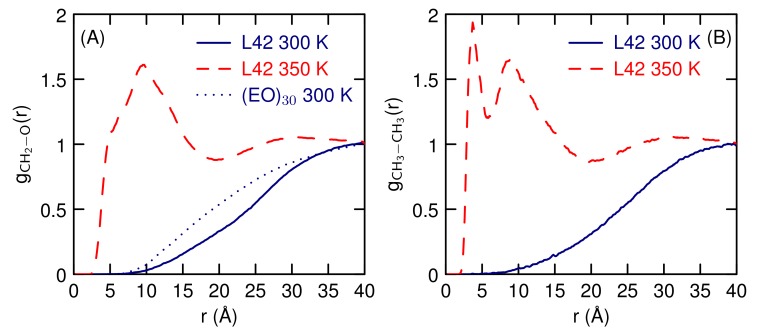
Radial distribution functions between atoms on different polymer strands for CH${}_{2}$–O (**A**) and CH${}_{3}$–CH${}_{3}$ (**B**) atoms.

The correlation functions between atoms of different polymers are shown on Figure 13. At 300 K, the CH${}_{2}$ carbons and the oxygen atoms do not get close to each other (consistent with Figure 8) and are a little farther apart for L42 than for (EO)${}_{30}$. At 350 K, the CH${}_{2}$–O and CH${}_{3}$–CH${}_{3}$ pairs get closer and come into direct contact. The largest peak, at a distance of 3.7 Å, is between CH${}_{3}$–CH${}_{3}$, indicating the significance of the hydrophobic interaction on the transition to the contact pair.

The changes in the number of nearest-neighbors between the contact pair and the separated pair for the L42 polymers, at a temperature of 350 K are given in Table 4. The cut-off distances used to determine nearest-neighbors are 5.0 Å, 5.0 Å, and 3.5 Å for CH${}_{3}$–CH${}_{3}$, CH${}_{3}$–O${}_{W}$, and O–O${}_{W}$, respectively. (This analysis is for the PO methyl groups only, not the terminal methyls.) In either state, there are a large number of contacts between the methyl groups, about 41, among the 22 PO methyl groups on L42. The contact pair can be made without much loss of the hydrophobic contacts present in the monomer. The contact pair makes 14 contacts between methyl groups on different chains, and one intra-chain contact is lost. There are also significantly fewer water-methyl group nearest neighbors in the contact pair. The decrease in oxygen-water neighbors is small, only 7 for The 60 oxygen atoms on the two L42 molecules. This data suggests that pairing involves increased contact between the methyl groups, some de-solvation of the methyl groups and no significant change the water structure near the oxygens.

## 4. Discussion

The newly developed course-grained model is able to capture the temperature response of the L42 block co-polymer. Previous CG studies have simulated micellization of Pluronic polymers, but not the temperature response [10] or simulated the temperature response of single PEO polymers of various lengths [16]. Here, we have demonstrated that our CG model can be used to study the temperature effects on aggregation between two polymer chains. While we have not established a value for the LCST, we find that at a temperature above the cloud point temperature, there exists a free energy minima for the a dimer pair in contact (Figure 9). At room temperature, there is no contact pair minimum and the polymers weakly repel each other. For (EO)${}_{30}$, there is no tendency for polymer strands to aggregate over the temperature range studied, in agreement with the lack of an LCST for this polymer. For a single polymer, there is no structural transition, just a gradual decrease in size (Figure 6) as temperature increases. This suggests that for polymers of this length, 30 monomers, The temperature response involves multiple polymers and not a conformational change of a single polymer.

Upon formation of the contact pair, there is a large change in entropy (Figure 10), with a decrease in −TS of −25 kcal/mol and big increase in enthalpy. The formation of the contact pair increases the number of methyl-methyl nearest neighbors, a decrease in the number of water-methyl neighbors, and little change in the polymer oxygen-water neighbors (Table 4). The gain in the number of hydrophobic contacts (13) is consistent with the large change in entropy. Song and Molinero, using similar CG models as used in this study, find that for the methane pair potential of mean-force The contact pair increases the entropy by 4.8 kcal/mol K${}^{-1}$ [23]. At 350 K, this gives −1.7 kcal/mol for −TS, which, when multiplied by 13, gives −22 kcal/mol, in good agreement with the entropy change found for the L42 polymers. This suggests that the association is driven by the hydrophobic interaction between the methyl groups on the PO units. It is interesting that mW water model [19], which lacks hydrogens and therefore any rotational contribution to the entropy of hydration, reproduces the entropy so well. The value of 4.8 kcal/mol K${}^{-1}$ is very close to the results using all-atom models [23].

A number of theoretical studies have studied the mechanisms of aggregation for PEO [54,55,56,57,58]. One proposed mechanism for the LCST properties of PEO involves a competition between water-water and water-ether oxygen hydrogen bonds [55,56,57]. As temperature increases, the polymer-water hydrogen bonds are weakened relative to water-water hydrogen bonds, perhaps due to changes in polymer backbone dihedral angles [55], and the solution phase separates. This mechanism predicts the LCST transition to be enthalpically driven, as opposed to the earlier entropically driven mechanism of Kjellander and Florin [54]. Ashbaugh and Paulaitis argue that is the hydrophobic nature of the carbon atoms that leads to phase separation [58]. Our results suggest that the Pluronic polymers follow a different mechanism from any of the PEO mechanisms. That the mechanisms are different is not that surprising considering the LCST for PEO is much higher and occurs only for longer polymers, and the PPO block contain a hydrophobic methyl group not present in PEO. In our proposed mechanism for L42, the hydrophobic interaction between these methyl groups is what drives aggregation, and the aggregation is entropically driven. Interestlngly, methyl groups may play a role even in PEO. The SANS experiments of Hammouda, et al, suggest that even for large polymers, the methyl groups on the chain ends promote aggregation, in a comparison with PEO capped with alcohol groups [59].

The LCST transition can be studied further by changing sequence, and changing the EO weight percent, and polymer length. Future studies can also simulate solutions with multiple polymers, to examine the aggregation process, and determine the value of the LCST. From these studies, the interactions which promote aggregation and the thermoresponsive nature of the polymers can be further characterized.

## Figures and Tables

**Table 1:** Two-body potential parameters for ether and previously published parameters for an alkane methyl group (A) [24] and water [19].

		σ (Å)	ϵ (kcal/mol)	a (Å)
CH${}_{3}$	CH${}_{3}$	3.98	0.11	1.8
CH${}_{2}$	CH${}_{2}$	3.98	0.11	1.8
CH${}_{3}$	CH${}_{2}$	3.98	0.11	1.8
CH${}_{3}$	O	3.14	0.40	2.0
CH${}_{2}$	O	3.14	0.40	2.0
O	O	4.10	0.10	1.8
CH${}_{3}$	H${}_{2}$O	3.65	0.44	1.8
CH${}_{2}$	H${}_{2}$O	3.54	0.44	1.8
O	H${}_{2}$O	2.20	4.20	1.8
CH${}_{3}$ (A)	CH${}_{3}$ (A)	4.64	0.100	1.8
CH${}_{3}$ (A)	H${}_{2}$O	4.25	0.165	1.8
H${}_{2}$O	H${}_{2}$O	2.3925	6.189	1.8

**Table 2:** Three-body potential parameters for ether and previously published parameters for water [19]. The central atom is given first.

			ϵ (kcal/mol)	λ	cos ($\theta_{0}$)
CH${}_{3}$	CH${}_{3}$	O	0.10	4.55	0.64
CH${}_{2}$	CH${}_{2}$	O	0.10	4.55	0.64
CH${}_{3}$	CH${}_{2}$	O	0.10	4.55	0.64
CH${}_{2}$	CH${}_{3}$	O	0.10	4.55	0.64
CH${}_{3}$	O	O	0.40	1.8	0.07
CH${}_{2}$	O	O	0.40	1.8	0.07
O	CH${}_{3}$	CH${}_{3}$	0.40	1.8	0.17
O	CH${}_{3}$	CH${}_{2}$	0.40	1.8	0.17
O	CH${}_{2}$	CH${}_{3}$	0.40	1.8	0.17
O	CH${}_{2}$	CH${}_{2}$	0.40	1.8	0.17
O	CH${}_{3}$	O	0.10	4.55	0.68
O	CH${}_{2}$	O	0.10	4.55	0.68
O	O	H${}_{2}$O	3.50	10.0	0.14
O	H${}_{2}$O	H${}_{2}$O	4.20	18.5	−1/2
H${}_{2}$O	CH${}_{3}$	CH${}_{3}$	0.44	10.0	−1/2
H${}_{2}$O	CH${}_{3}$	CH${}_{2}$	0.44	10.0	−1/2
H${}_{2}$O	CH${}_{2}$	CH${}_{2}$	0.44	8.0	−1/2
H${}_{2}$O	O	O	4.20	10.0	−1/3
H${}_{2}$O	H${}_{2}$O	O	6.189	20.0	−1/2
H${}_{2}$O	H${}_{2}$O	H${}_{2}$O	6.189	23.15	−1/3

**Table 3:** Properties of liquid 1,2-dimethoxyethane and the aqueous solvation thermodynamics values of 1,2-dimethoxyethane, at 298 K.

	Model	Experiment
density (g/cm${}^{3}$)	0.864 ± 0.002	0.861
ΔH${}_{vap}$ (kcal/mol)	8.2 ± 0.2	8.79
surface tension (nm/m)	26 ± 2	23.9
ΔG${}_{solv}$ (kcal/mol)	−6.4 ± 0.1	−4.8
ΔH${}_{solv}$ (kcal/mol)	−13.5 ± 0.6	−14.2
ΔS${}_{solv}$ (cal/(mol K))	−24 ± 2	−31.5

**Table 4:** The number of nearest-neighbors, for The L42 polymers when in contact and when separated, at 350 K.

	CH${}_{3}$–CH${}_{3}$		CH${}_{3}$–O${}_{W}$	O–O${}_{W}$
	Intra-Chain	Inter-Chain		
contact pair	41 ± 1	14 ± 1	286 ± 4	39 ± 1
separated pair	42 ± 1	0	363 ± 3	46 ± 1
difference	−1 ± 1	14 ± 1	−77 ± 5	−7 ± 1

## Abbreviations

The following abbreviations are used in this manuscript:

CG	coarse-grained
DME	dimethoxyethane
EO	ethylene oxide
PEG	polyethylene glycol
PEO	polyethylene oxide
PO	propylene oxide
PPO	polypropylene oxide
L42	(EO)${}_{4}$-(PO)${}_{22}$-(EO)${}_{4}$
H	enthalpy
S	entropy
w	potential of mean force
